# The Regulatory Role of RNA Metabolism Regulator TDP-43 in Human Cancer

**DOI:** 10.3389/fonc.2021.755096

**Published:** 2021-10-28

**Authors:** Xueyou Ma, Yufan Ying, Haiyun Xie, Xiaoyan Liu, Xiao Wang, Jiangfeng Li

**Affiliations:** ^1^ Department of Urology, The First Affiliated Hospital, Zhejiang University School of Medicine, Hangzhou, China; ^2^ Cancer Center, Zhejiang University, Hangzhou, China; ^3^ Department of Pathology, The First Affiliated Hospital, Zhejiang University School of Medicine, Hangzhou, China

**Keywords:** TDP-43, mRNA metabolism, ncRNA, cancers, epigenetics

## Abstract

TAR-DNA-binding protein-43 (TDP-43) is a member of hnRNP family and acts as both RNA and DNA binding regulator, mediating RNA metabolism and transcription regulation in various diseases. Currently, emerging evidence gradually elucidates the crucial role of TDP-43 in human cancers like it is previously widely researched in neurodegeneration diseases. A series of RNA metabolism events, including mRNA alternative splicing, transport, stability, miRNA processing, and ncRNA regulation, are all confirmed to be closely involved in various carcinogenesis and tumor progressions, which are all partially regulated and interacted by TDP-43. Herein we conducted the first overall review about TDP-43 and cancers to systematically summarize the function and precise mechanism of TDP-43 in different human cancers. We hope it would provide basic knowledge and concepts for tumor target therapy and biomarker diagnosis in the future.

## Introduction

TAR-DNA-binding protein-43 (TDP-43, also named TARDBP) was first cloned (43 kDa) in 1995, and its name was derived from the characteristics of binding to human immunodeficiency virus type 1 (HIV-1) TAR DNA sequence motifs (1). With the profound recognition of molecular structure, the RNA binding role of TDP-43 was gradually uncovered in addition to its DNA binding function. As a member of hnRNP family, TDP-43 has been confirmed to regulate mRNA splicing, mRNA transport, mRNA stability, and pri-miRNA processing (2–7). Emerging evidence has demonstrated that TDP-43 was one of the crucial RNA binding proteins to be involved in diverse diseases by mediating the RNA metabolisms. The best-studied disease about TDP-43 is neurodegeneration: abnormal RNA processing induced by gains of toxic properties and losses of normal TDP-43 functions leading to neurodegeneration (8–10). Numerous researches have confirmed that RNA metabolisms and non-coding RNA (ncRNA) regulations are closely involved in carcinogenesis and tumor progressions (11–15). As the important intermediate regulator, RNA binding protein (RBP) TDP-43 has been widely researched and well recognized. Recently, accumulating studies have indicated the crucial regulatory mechanisms of TDP-43 in various cancers. Herein we will focus on the specific role of TDP-43 in different human cancers to provide an in-depth understanding of TDP-43 in tumor mechanism research, clinical detection, and therapy.

## Structure of TDP-43 and Mutations

### Domain Structure of TDP-43 for RNA Binding Function

**Figure 1:**
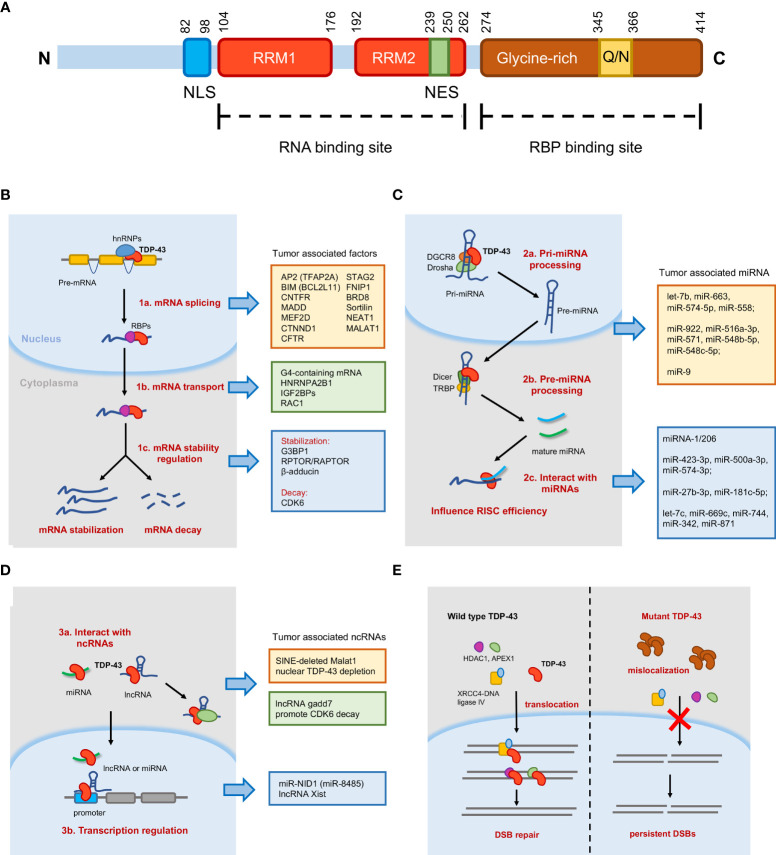
Schematic diagram of TAR-DNA-binding protein-43 (TDP-43) domain structure and molecular mechanisms mediated by TDP-43. **(A)** TDP-43 protein contains 414 amino acids, an N-terminal domain (NTD), two RNA recognition motif (RRM1 and RRM2) domains, a glutamine/asparagine-rich (Q/N) and glycine-rich C-terminal region, and two singles (bipartite nuclear localization signal and nuclear export signal). **(B)** In the nucleus, TDP-43 is essential for messenger RNA (mRNA) splicing and transport. In the cytoplasm, TDP-43 regulates mRNA stability, including mRNA stabilization and mRNA decay. **(C)** TDP-43 participates in microRNA (miRNA) processing as well as interacts with mature miRNAs to influence RNA-induced silencing complex (RISC) efficiency. **(D)** TDP-43 could interact with long non-coding RNA (lncRNA) or miRNA to regulate transcription. TDP-43 could also bind lncRNA to regulate target proteins. **(E)** Model of TDP-43 function in DNA damage repair. In normal cells, TDP-43 helps in the nuclear import of double-strand break (DSB) repair proteins and stimulates DSB repair. The mutant TDP-43 (Q331K, A382T) reduces mislocalization in the cytoplasm and abrogates the nuclear localization of DSB repair proteins, resulting in the accumulation of DSBs and upregulated DNA damage response.

Human TDP-43 is located in chromosome 1p36.22, belonging to a member of hnRNP family (16). As illustrated in **Figure 1A**, 414 amino acid composed the TDP-43 protein that contains an N-terminal domain (NTD), two DNA/RNA recognition motif (RRM1 and RRM2) domains, a glutamine/asparagine-rich (Q/N) and glycine-rich C-terminal region, and two signals (bipartite nuclear localization signal, NLS, and nuclear export signal, NES). For the cellular expression pattern, TDP-43 is frequently relatively less expressed in the cytoplasm than in the nucleus, which indicates the nuclear RNA binding regulatory role (17). Two RRMs are direct RNA-binding domains by characteristically recognizing UG repeats of single-stranded RNA, and the binding affinity increases with the number of repeats (18–20). However, non-UG repeat sequences were also reported, which broadened the RNA binding targets and complexity of regulations (21). As previous individual nucleotide resolution CLIP (iCLIP) sequencing data revealed, a large proportion of transcriptomes was detected; most binding sites are mapped to introns, lncRNAs, and intergenic transcripts, which had greatest enrichment of UG-rich motifs (4, 22). In terms of the glycine-rich C-terminal region, it provides the region spanning residues 321 to 366 to act as the interaction site with other RBPs, including hnRNP family like hnRNPA2B1 and Ubiquilin-2 (23, 24).

### Pathogenic TDP-43 Mutations

More than 50 disease-associated mutations of TDP-43 gene have been identified in amyotrophic lateral sclerosis (ALS) or frontotemporal lobar degeneration (FTLD) patients, which are mainly concentrated in the glycine-rich C-terminal region, indicating that the ability of TDP-43 for cooperative assembly on RNA binding sites plays a role in disease mechanisms. TDP-43 mutation (Q331K) resulted in cytosolic mislocalization, impairing the nuclear localization of XRCC4–DNA ligase 4, which contributed to persistent DNA damage accumulation (25). TDP-43 mutation (A382T) disrupted the dynamics of stress granules (SGs), reducing the ability of cells to respond to stress (26). Additionally, mislocalization of A382T mutation resulted in transcription-dependent R-loop accumulation and DNA replication defects (27). A recent study demonstrated that TDP-43 ALS-associated mutants (A315T, Q331K, M337V) resulted in DNA damage through inducing cytoplasmic mislocalization and SG formation (28). In addition, mutations in NTD (L27A, L28A, V31R, and T32R) reduced the splicing activity of TDP-43 and induced mislocalization and accumulation through destabilizing the NTD (29). Mutations are also quite frequent in various types of cancer and have been recognized as the driving force for carcinogenesis and progression. However, the mutation condition of TDP-43 in tumors is still unelucidated. More attention is needed to focus on the connection between TDP-43 mutation and dysregulation in tumors.

## Tumor-Associated RNA Metabolisms Mediated by TDP-43

Generally, TDP-43 is well confirmed by previous studies to mainly regulate RNA metabolism, like biogenesis, processing, decay, and transport, in various diseases. Here we stated these tumor-associated molecular mechanisms with several parts in detail as discussed in the following paragraphs (**Figures 1B–D**).

### TDP-43-Mediated mRNA Splicing

Tollervey et al. used iCLIP sequencing to identify the TDP-43-mediated mRNA splicing events in ALS and FTLD (4). Total splicing changes in 158 alternative cassette exons were identified in TDP-43 knock-down experiments, and among all the splicing events, several proteins encoded by the alternative mRNA isoforms regulated by TDP-43 were found to be involved in the development of various tumors, such as TFAP2A (30), BCL2L11 (31), CNTFR (32), MADD (33), MEF2D (34), and CTNND1 (35). TDP-43 was also reported to bind to CFTR pre-mRNA promoting the skipping of exon 9 (19), which functions as a tumor suppressor in colorectal cancer (36). In another study, De Conti et al. identified six *bona fide* splicing events directly induced by TDP-43 through high-throughput sequencing and HTS-based splicing junction analysis, and they found that the isoform expression levels of four genes were changed significantly (37). Previous studies showed that these four genes [MADD (38), STAG2 (39), FNIP1 (40), and BRD8 (41)] were potentially important for tumors. In mice models, abnormal TDP-43 function, owing to aggregation or disease-associated mutation, induced the wrong splicing of trafficking receptor sortilin (42), the loss of which promoted cell proliferation of lung cancer cells (43). Similarly, TDP-43 also regulated lncRNA expression. Tollervey et al. also found that tumor-associated lncRNA NEAT1 and MALAT1 were significantly enriched by TDP-43, indicating the importance of TDP-43 in lncRNA expression (4). Further confirmation was reported in a recent research; TDP-43 enhanced the polyadenylated short isoform of NEAT1 to promote cell pluripotency (44). In contrast to the splicing promotion of TDP-43 on conserved exons, TDP-43 repressed the splicing of non-conserved cryptic exons to maintain intron integrity (45). Consistently, another recent study also found that TDP-43-mediated splicing repression protected the transcriptome by preventing aberrant splicing (46) (**Figure 1B**).

### TDP-43-Mediated mRNA Transport

The mRNA transport process is critical for physiological processes as well as cancer genesis and maintenance. TDP-43 was reported to bind and transport G-quadruplex-containing mRNAs into neurites for local translation (47). G-quadruplex (G4) was known to influence RNA post-transcriptional mechanisms, thereby impacting neurodegenerative disease and cancer (48). In addition, TDP-43 tended to regulate mRNA transport with other RBPs. Freibaum et al. identified 126 proteins that were exclusively in association with TDP-43 to co-regulate mRNA transport and stability in HEK-293 cells (49), including HNRNPA2B1 and IGF2BPs. A recent study revealed that TDP-43 regulated the anterograde and retrograde transport of Rac1 messenger ribonucleoprotein with two other RNA-binding proteins, FMRP and Staufen1, respectively, in mouse neuronal dendrites (50) (**Figure 1B**). As reported recently, a novel miR-NID1 (miR-8485) transcribed from NRXN1 intron 5 could interact with AGO and together transported by TDP-43 to the nucleus for further regulation (see the details in **Figure 1C**) (51). These genes have been confirmed by previous studies to be crucial for various cancers.

### TDP-43-Mediated mRNA Stability

The 3′ UTR binding capability of TDP-43 accounts for mRNA stability regulation (**Figure 1B**). Moreover, 34% 3′ UTR binding in the cytoplasm, 3.2% in the nuclear fraction, and 3.8% in total cell extract of SH-SY5Y cell were identified in the iCLIP analysis of RNAs bound by TDP-43, which indicated the mRNA stability potential regulation of TDP-43 (4). TDP-43 accumulation was recognized to contribute to RNA instability. A finding from recent research indicated that TDP-43-EGFP overexpression altered the RNA stability of 1,330 transcripts with at least ≥1.5-fold change, and 75% (1,002 transcripts) were destabilized (52). That means TDP-43 can regulate stability balance in two aspects: maintain mRNA stability or promote mRNA decay. In the aspect of stability maintenance, a case reported that TDP-43 stabilizes G3BP1 transcripts by targeting a highly conserved cis-regulatory element in the 3′ UTR (53). Another report also illustrated that TDP-43 mediated MTORC1-TFEB signaling by partially stabilizing RPTOR/RAPTOR to regulate autophagy (54). TDP-43 also regulated β-adducin (Add2) expression levels by increasing Add2 mRNA stability in the brain (55). By contrast, in the aspect of mRNA decay, CDK6 expression at mRNA and protein levels was both inhibited by TDP-43 *via* regulating the pRb cell cycle pathway. At the molecular level, larger introns and the 3′ UTR of CDK6 pre-mRNA contained a (GU)25 sequence. However, the precise mechanism of TDP-43 and CDK6 mRNA was not elucidated at that time (56). Afterward, a report confirmed that UV-induced lncRNA gadd7 directly interacted with TDP-43 and promoted CDK6 mRNA decay (57) (**Figure 1D**). All the above-mentioned genes have been demonstrated to play crucial roles in cancers.

### TDP-43-Mediated miRNA Processing

As an important class of ncRNAs in cancers, miRNA biogenesis and processing are complex processes involved with various RBPs, including TDP-43 (**Figure 1C**). TDP-43 was found to promote miRNA biogenesis in both pri-miRNA processing (nucleus, pri-miRNA to pre-miRNA) and pre-miRNA processing (cytoplasm, pre-miRNA to mature miRNA) steps (6). To be specific, in cellular nucleus, TDP-43 directly bound to Drosha to compose the miRNA processor together and mediated the transfer from pri-miRNA to pre-miRNA, and in the cytoplasm TDP-43 continuously promoted the processing of these pre-miRNAs to mature miRNAs *via* interacting with Dicer complex and binding to their terminal loops (6). Previous studies showed that TDP-43 processed a lot of tumor-associated miRNAs. The first report of miRNA changes in Hep‐3B cells after TDP-43 depletion indicated that TDP‐43 has the potential to affect the levels of four miRNAs (let‐7b, miR‐663, miR‐574‐5p, and miR‐558 ) by potentially binding to their sequence and/or precursor element (58). Several studies reported altered miRNA expressions corresponding with dysregulated TDP-43. Significantly dysregulated microRNAs (miR-922, miR-516a-3p, miR-571, miR-548b-5p, and miR-548c-5p) in frontotemporal lobar degeneration with TDP-43 pathology were caused by progranulin mutations (59). The levels of miR-9 and its precursor pri-miR-9-2 decreased in downregulated TDP-43 of FTD/ALS patients (60). Recently, the TDP-43-processed miRNAs in tumors were also uncovered (see details below) (15). Interestingly, this research reported additional functions mediated by TDP-43, like the regulatory role of TDP-43 in isoforms of miRNA (isomiR) pattern and miRNA arm selection, which needs further investigation for precise mechanisms.

### The Interaction Between TDP-43 and Mature miRNA and lncRNA

In contrast to RNA metabolism like mRNA splicing and miRNA processing, more research proposed the direct interaction between TDP-43 and mature miRNAs or lncRNA to cooperatively regulate downstream, which further extends the function and mechanism complexity of TDP-43. Several tumor-associated miRNAs and lncRNAs have been confirmed to interact with TDP-43. To be specific, TDP-43 selectively disrupted miRNA-1/206 incorporation into the RNA-induced silencing complex to dampen the miRNA target decay efficiency, suggesting a processing-independent mechanism for the differential regulation of miRNA activity (61) (**Figure 1C**). A novel miR-NID1 (miR-8485) transcribed from NRXN1 intron 5 could interact with AGO and be transported by TDP-43 to the nucleus and combined together to directly bind to the promoter region of NRXN1, reversely inhibiting its linear production (51) (**Figure 1D**). As reported in lung cancer cells, TDP-43 could also directly interact with mature miRNAs (miR-423-3p, miR-500a-3p, and miR-574-3p) (15). Recently, Hawley et al. found a negative feedback network between TDP-43 and miR-27b-3p/miR-181c-5p, which is dependent on TDP-43 nuclear localization (62). In addition, Fu et al. revealed that TDP-43 could selectively bind a large group of miRNAs *via* RRM1 in N2a cells, such as let-7c, miR-669c, miR-744, miR-342, and miR-871 (63) (**Figure 1C**).

In terms of lncRNA, interaction with RBPs is a well-studied mechanism for scaffold lncRNAs. As mentioned above, TDP-43 combined with lncRNA gadd7 promotes CDK6 decay under UV treatment (57). Recently, a study revealed that the short interspersed nuclear element (SINE)-deleted Malat1 will bind stronger with TDP-43 and cause nuclear TDP-43 depletion and subsequent TDP-43 aggregation (64). Another study showed that the lncRNA Xist-TDP-43 assembly is essential to the anchoring of Xist to the inactive X (Xi) compartment and heritable gene silencing (65) (**Figure 1D**). All these indicated the complicated regulation network of TDP-43 with ncRNAs, which needs further investigation.

### The Interaction Between TDP-43 and Other RBPs

The glycine-rich C-terminal region of TDP-43 is regarded as the structural basis for the interaction between TDP-43 and other RBPs (66), which improved the regulation complexity of TDP-43. As a member of hnRNP family, TDP-43 was well confirmed to cooperate with other hnRNP family proteins like HNRNPA2B1 (23, 67). Among the co-immunoprecipitation of TDP-43 in HEK-293 cells, 126 proteins were identified exclusively to be in association with TDP-43, which were enriched in two clusters (nuclear splicing cluster and cytoplasmic translation cluster) (49). Another study also reported similar results; hnRNP L, PTB/nPTB, and hnRNP A1/A2 interacted with TDP-43 to inhibit the production of a truncated human SORT1 receptor in neurodegenerative diseases (68). Recent tumor-associated research found that the TDP-43/SRSF3 complex controlled specific splicing events of downstream genes PAR3 and NUMB to promote the progression of triple-negative breast cancer (69). Concerning the significance of RNA metabolisms in tumor progression, the network mediated by TDP-43 combined with other RBPs has potential influence.

## The Cellular Biological Events Induced by the TDP-43 Network

Through literature review and the context presented above, the cellular biological events mediated by the TDP-43 network mainly include cell cycle, apoptosis, and autophagy (37, 54, 57, 70). It mainly depends on the target RNAs of TDP-43 in various diseases. All the above-mentioned biological events were vital for carcinogenesis and tumor progressions. In addition, other biological events induced by TDP-43 also deserve attention.

### Liquid–Liquid Phase Separation

Recently, the liquid–liquid phase separation (LLPS) of TDP-43 revealed a novel mechanism by regulating RNA stability and ribonucleoprotein assembly in certain diseases. For structure basis, α-helical component in the center (residues 320–340) of the C-terminal domain is related to the self-association of the protein and LLPS (71, 72); another research found that phosphomimetic substitution at S48 of NTD disrupted TDP-43 LLPS (73). In addition, a recent study revealed acetylation and HSP70-regulated TDP-43 phase separation and conversion into solid phase (74). LLPS has been found to be involved in several diseases, especially neurodegenerative diseases. It is an emerging area for LLPS in tumors, and its important regulatory role comes to the realization of scientists. One recent paper suggested that mutations in the tumor suppressor SPOP were linked to specific phase separation defects, which induced the upregulation of various proto-oncogenic proteins targeted by normal SPOP-mediated protein proteasomal degradation (75).

### DNA Damage and Nonhomologous DNA End Joining

The role of TDP-43 in DNA damage and repair could also not be neglected (**Figure 1E**). Neurodegenerative diseases are known to harbor the accumulation of damaged DNA as well as impairment in DNA repair mechanisms. Mislocalization of TDP-43 impairs the nuclear localization of DNA double-strand break (DSB) repair proteins (such as HDAC1 and APEX1) and contributes to the accumulation of DNA damage, thus promoting cell death (76–78). In addition, TDP-43 deletion and mislocalization of mutated TDP-43 (A382T) induced transcription-dependent R-loop accumulation and resulted in DNA replication defects (27, 79). Nonhomologous DNA end joining (NHEJ) is one of the major DSB repair pathways in eukaryotes. TDP-43 has recently been found to function as an accessory factor during NHEJ (28, 80). In a proteomics study in 2010, TDP-43 was found to interact with Ku70, a core factor initiating the NHEJ pathway (49). Mitra et al. observed a direct interaction between recombinant TDP-43 and purified XRCC4–DNA ligase IV complexes, even in the absence of DNA (81), which helps in the nuclear transport of the XRCC4–DNA ligase IV complex. Their subsequent study revealed that TDP-43 mutation (Q331K) enhanced cytosolic mislocalization, preventing the nuclear translocation of XRCC4–DNA ligase IV and thus resulting in persistent DSBs and upregulated DNA damage response (25). These results established TDP-43 as a new regulating factor for NHEJ in neuronal cells. However, further investigations are required to examine whether TDP-43 has a potential role in DNA damage to regulate carcinogenesis and tumor progression.

## The Potential Role of TDP-43 in Cancers

By reviewing all the literature about TDP-43 and cancers, we totally found 11 types of cancers involved. In this part, we will review this topic by cancer type in detail (**Table 1**).

**Table 1:** The potential role of TDP-43 in cancers.

Tumor	Dysregulation	Associated factor	Downstream	Mechanism	Biological function	Function	Ref./PMID
Breast cancer	–	Curcumin	**-**	**-**	Promoted cell apoptosis	Tumor suppressor	21239154
Breast cancer	–	–	–	TDP-43 35 kDa fragment	Promoted cell apoptosis	Tumor suppressor	29421661
Breast cancer	Downregulated	TRIM16	CDK6, E2F1, pRb	mRNA stability	Inhibited cell cycle	Tumor suppressor	26902425
Triple-negative breast cancer	Upregulated	SRSF3	PAR3, NUMB	mRNA splicing	Promoted proliferation and metastasis	Oncogenic factor	29581274
Lung cancer	–	miR-423-3p	CRK, LCP2, ITGA9	miRNA interaction	Promoted cell migration	Oncogenic factor	28952053
	–	miR-500a-3p	LIF, PAPPA	miRNA processing and interaction	Inhibited tumors	Tumor suppressor	28952053
Non-small cell lung cancer, NSCLC	Upregulated	MALAT1	ABCA1, LPHN2	lncRNA stability	Promoted cell proliferation and migration	Oncogenic factor	26265046
Lung cancer	Downregulated	FasL	–	mRNA stability	Promoted cell apoptosis	Tumor suppressor	31978067
Glioblastoma	Upregulated	HDAC6	–	mRNA stability	Activated autophagy, suppressed stress-induced apoptosis	Oncogenic factor	28915616
Neuroblastoma	Downregulated	TRIM16	CDK6, E2F1, pRb	mRNA stability	Inhibited cell cycle	Tumor suppressor	26902425
Neuroblastoma	–	–	–	–	–	Good prognosis indicator	30394813
Neuroblastoma	–	β-N-methylamino-L-alanine	–	TDP-43 truncated forms (C-terminal fragments), phosphorylated and high molecular weight forms of TDP-43	–	–	23665941
Hepatocellular carcinoma	Upregulated	miR-520 family	PFKP	Transcription inhibition	Promoted glycolysis and proliferation	Oncogenic factor	23389994
Hepatocellular carcinoma	Upregulated	GSK3β	Wnt/β-catenin signaling pathway	mRNA translation	Promoted proliferation and metastasis	Oncogenic factor	33163270
Melanoma	Upregulated	–	GLUT4	–	Promoted proliferation and metastasis	Oncogenic factor	27786596
Cervical cancer (Hela cell)	–	p53	–	–	Induced p53-dependent G2/M arrest and p53-independent cell death	Tumor suppressor	22133803
Cervical cancer (Hela cell)	–	–	–	DNA damage	Prevented R loops accumulation and DNA damage	–	33301444
Ewing sarcoma	–	–	–	Mutation rs9430161	Higher tumor susceptibility	–	22327514
Leukemic (pre-B-ALL cell line MHH-CALL3 cell)	Upregulated	–	–	–	–	–	21783252
Prostate cancer	Downregulated	–	–	–	–	Tumor biomarker	31404106
Colon cancer	–	1,25(OH)2D3	–	–	–	Tumor suppressor	21864731

### Breast Cancer

Breast cancer is the most and best investigated cancer on TDP-43 function and mechanism. The role of TDP-43 in breast cancer was first mentioned in curcumin therapy research. Investigators found that the anticancer agent curcumin could effectively inhibit breast cancer cell proliferation (cell cycle arrest and apoptosis); further proteomic analysis suggested that TDP-43 was significantly downregulated after curcumin treatment (82), but the specific molecular mechanism of how TDP-43 is involved was not explained. In another similar research, this issue was solved to some degree. In this study, the authors found that the cleaved TDP-43 (35-kDa fragment) mediated by caspase 3 was cytotoxic and promoted breast cancer cell apoptosis, which can serve as a therapeutic target to treat breast cancer (83). Another study revealed that TDP-43 was required for TRIM16-induced cell growth inhibition of breast cancer and neuroblastoma and suggested TDP43 as a good prognosis indicator (84). Ke et al. found that TDP-43 was significantly upregulated in triple-negative breast cancer (TNBC) compared with normal tissue. Further biofunction assay and mRNA sequencing analysis confirmed that TDP-43 regulated TNBC unique alternative splicing events to promote tumor progression. Through immuno-precipitation and mass spectrometry analysis, SRSF3 was finally identified as the co-regulator with TDP-43 to mediate the alternative splicing of several targets, including genes PAR3 and NUMB (69).

### Lung Cancer

Two studies suggested that TDP-43 regulated lung cancer progression involving miRNAs and lncRNA. To investigate the mechanism of TDP-43 in regulating lung cancer-associated miRNA biogenesis, Chen et al. used miRNA sequencing after knocking down TDP-43 and found that TDP-43 knockdown affected the expression of many miRNAs and altered the patterns of different isoforms of miRNAs (isomiRs) and miRNA arm selection. In addition, TDP-43 was also confirmed to directly interact with some mature miRNAs like miR-423-3p and miR-500a-3p. Further analysis showed that miR-423-3p was responsible for the cell migration induced by TDP-43, and miR-500a-3p may serve as a prognostic marker of lung cancer (15). All the above-mentioned findings indicated the crucial miRNA regulating role of TDP-43 in lung cancer progression. In terms of lncRNA, TDP-43 could also bind the 3′ UTR of MALAT1 to maintain its stability in lung cancer; the knock-down of TDP-43 significantly suppressed cell proliferation and migration by downregulating the MALAT1 level (85). One recent report proposed a little controversy in the aspect of TDP-43 and apoptosis, and it suggested that TDP-43 restored the sensitiveness of lung cancer cells to cisplatin or lipopolysaccharide by protecting the apoptotic inducer FasL mRNA from decay (86). Maybe the different cell biological processes vary in the different targets of TDP-43.

### Glioblastoma and Neuroblastoma

It is not hard to understand the role of TDP-43 in nerve system tumors like glioblastoma and neuroblastoma, considering the vital pathological factor role of TDP-43 in neurodegenerative diseases. In glioblastoma, TDP-43 overexpression, induced by tumor cell nutrition deprivation, promoted tumor progression. The elevated TDP-43 could stabilize HDAC6 by binding to 3′ UTR, consequently promoting survival *via* activating autophagy (87). In terms of neuroblastoma, two studies have regarded TDP-43 as a beneficial prognosis predictor, and a high expression of TDP-43 indicated a good prognosis in neuroblastoma patients (84, 88). For molecular mechanism, scientists speculated that TDP-43 combined with tumor suppressor TRIM16 to regulate CDK6 stability and, consequently, the E2F1/pRb cell cycle pathway (84). However, regardless of the total amount of TDP-43, another paper also proposed the importance of altered TDP-43 truncated forms (C-terminal fragments), phosphorylated and high molecular weight forms of TDP-43, in the carcinogenesis of neuroblastoma. These forms of TDP-43 could be induced by neurotoxic amino acid β-N-methylamino-L-alanine, which was produced by most cyanobacteria and extensively distributed in different environments all over the world (89).

### Hepatocellular Carcinoma

The upregulation of TDP-43 was confirmed to act as a glycolysis regulator in hepatocellular carcinoma (HCC). Knockdown of TDP-43 significantly inhibited HCC cell proliferation; through mRNA sequencing and bioinformatic sequence analysis, TDP-43 could directly bind to the promoter of miR-520 family to inhibit the expression. In addition, PFKP was further identified as the direct target of the miR-520 family. Consequently, the TDP-43/miR-520s/PFKP regulatory axis was established to explain the HCC glycolysis mechanism (90). Recently, another study confirmed the significance of TDP-43 in promoting HCC cell proliferation and metastasis by suppressing GSK3β protein translation and subsequent Wnt/β-catenin pathway activation (91).

### Melanoma

Similar to the regulatory role of TDP-43-mediated glycolysis in HCC, TDP-43 was also confirmed to be a novel oncogene promoting melanoma cell proliferation and migration, which was also a poor prognosis indicator for overall survival. For mechanism investigation, TDP-43 regulated the GLUT4 expression in an indirect way; however, the specific mechanism needed further investigation (92).

### Cervical Cancer

A preliminary influence test of TDP-43 on cervical cancer was also reported. In HeLa cells, overexpression of TDP-43 caused partially p53-dependent G2/M arrest and p53-independent cell death (93). Another study revealed that TDP-43 depletion increased the R-loop accumulation and associated genome instability in HeLa cells (27). More investigations of TDP-43 in cervical cancer are needed.

### Other Cancers

Several cancers only reported a few about the expression pattern of TDP-43, which indicates a potential regulatory function in tumorigenicity. Mutation rs9430161 [*P* = 1.4 × 10−20; odds ratio = 2.2), located 25 kb upstream of TDP-43, was associated with susceptibility to Ewing sarcoma. The variant was associated with TDP-43 expression levels (94). To screen the differentially expressed nucleolar proteins in leukemic cell lines with dimensional difference gel electrophoresis analyses, TDP-43 was found to be strongly expressed in the nucleoli of the leukemic pre-B-ALL cell line MHH-CALL3, suggesting that its identification effect differentiates various leukemia subtypes (95). The biomarker value of TDP-43 was also reported in prostate cancer. TDP-43, as a member of the detection panel, improved the detection rate with a magneto-nanosensor assay for serum circulating autoantibodies. Human serum samples from 99 patients (50 with non-cancer and 49 with clinically localized prostate cancer) were evaluated in this study. The area under the curve of TDP-43 receiver operating characteristic curves was 0.793 (95%CI, 0.512–1.00) (96). 1α,25-Dihydroxyvitamin D3 [1,25(OH)2D3] was reported to be a potential anticancer agent for various cancers, including colon cancer. To explore the mechanism of 1,25(OH)2D3 action on human colon cancer cells, a proteomic analysis was conducted, and it revealed that a large group of identified proteins, including SFPQ, SMARCE, KHSRP, TDP-43, and PARP1, were involved in RNA processing or modification in colon tumorigenicity (97).

## Conclusion

As a member of hnRNP family RBP, TDP-43 plays a significant role in RNA metabolism in various diseases, especially the cancers discussed here. The complex regulation network composed of mRNA active splicing, transport, stability, miRNA processing, and lncRNA interaction comes into focus and is realized by scientists for investigating the mechanisms of carcinogenesis and tumor progression. All the above-mentioned reviews indicate us the important role of TDP-43 involved in various cancers. We can conclude it in several aspects: (1) TDP-43-mediated important oncogene or tumor suppressor mRNA splicing and stability regulation, (2) the interaction between TDP-43 and miRNAs (miRNA processing or co-regulation), and (3) regulating downstream targets that interacted with lncRNA or other RBPs. We hope that this conclusion would provide some research experience or any ideas for other researchers who want to investigate this field. However, a small shortcoming is that only some preliminary findings indicate that TDP-43 is rather important in certain tumors, and the precise molecular mechanisms mediated by TDP-43 are still unclear, which need further investigation in the future. Considering the dysregulated expression pattern, it is also promising to find the target drug or translate it into a sensitive and specific biomarker for tumor therapy and diagnosis. To conclude, we hope that this review could provide detailed and systematic concepts and knowledge of cancer-associated TDP-43.

## Author Contributions

XM, YY, and HX performed literature search and reviewed the literature. XM drafted the manuscript. JL, XW, and XL revised and finalized the manuscript. All authors contributed to the article and approved the submitted version.

## Funding

This study was supported by the National Natural Science Foundation of China (nos. 81802564 and 82103243), the Zhejiang Province Medical and Health Scientific Research Project (2019RC033), the Zhejiang Provincial Natural Science Foundation of China (LY20H160022), and the China Postdoctoral Science Foundation (2020M681885).

## Conflict of Interest

The authors declare that the research was conducted in the absence of any commercial or financial relationships that could be construed as a potential conflict of interest.

## Publisher’s Note

All claims expressed in this article are solely those of the authors and do not necessarily represent those of their affiliated organizations, or those of the publisher, the editors and the reviewers. Any product that may be evaluated in this article, or claim that may be made by its manufacturer, is not guaranteed or endorsed by the publisher.
